# Micro-Lensed Negative-Curvature Fibre Probe for Raman Spectroscopy

**DOI:** 10.3390/s21248434

**Published:** 2021-12-17

**Authors:** Karolina Milenko, Stephanos Yerolatsitis, Astrid Aksnes, Dag Roar Hjelme, James M. Stone

**Affiliations:** 1 Department of Electronic Systems, Norwegian University of Science and Technology, O.S. Bragstads Plass 2b, 7034 Trondheim, Norway; astrid.aksnes@ntnu.no (A.A.); dag.hjelme@ntnu.no (D.R.H.); 2 SINTEF Microsystems and Nanotechnology, Gaustadalleen 23C, 0737 Oslo, Norway; 3 Department of Physics, University of Bath, Bath BA2 7AY, UK; s.yerolatsitis@bath.ac.uk (S.Y.); py1jms@bath.ac.uk (J.M.S.)

**Keywords:** raman sensing, hollow-core fibre, fibre probe

## Abstract

We developed a novel miniature micro-lensed fibre probe for Raman spectroscopy. The fibre probe consists of a single negative-curvature fibre (NCF) and a spliced, cleaved, micro-lensed fibre cap. Using a single NCF, we minimized the Raman background generated from the silica and maintained the diameter of the probe at less than 0.5 mm. In addition, the cap provided fibre closure by blocking the sample from entering the hollow parts of the fibre, enabling the use of the probe in in vivo applications. Moreover, the micro-lensed cap offered an improved collection efficiency (1.5-times increase) compared to a cleaved end-cap. The sensing capabilities of the micro-lensed probe were demonstrated by measuring different concentrations of glucose in aqueous solutions.

## 1. Introduction

There is a large and unmet need for real-time continuous detection and monitoring of complex analytes for applications in medicine, biology, chemistry, and environmental science. Optical fibre sensors for in vivo sensing have gained significant interest in the last three decades. Small-diameter optical fibre probes enable in vivo remote and real-time detection in biomedical diagnostics [1]. Raman spectroscopy is a minimally invasive analytical technique with high sensitivity and specificity. It was previously demonstrated that Raman spectroscopy can be used in a range of applications including the identification of gases [2,3,4], liquids [5,6], and drugs [7,8]. Combining Raman spectroscopy with optical fibres has also been widely researched [9,10,11]. Nevertheless, the use of solid-core optical fibres for Raman spectroscopy is restricted due to the strong broad background Raman signal generated by a solid silica core. The background increases linearly with fibre length [12] and may overwhelm the Raman signal from the sample. Therefore, optical fibre Raman probes normally use multiple fibres, distal optics, and filters to filter out the background [9]. This results in probe diameters too large (>1 mm) for many in vivo applications. In addition, most fibre Raman probes excite and collect the Raman signal from a small volume of the sample beneath the distal end of the probe [13]. As the Raman process is inherently weak, it is important to maximize the collection efficiency of the probe. Micro-lensed solid-core fibres have been proposed in [14,15] to increase the collection efficiency of the Raman probes without significantly increasing the outer diameter of the probe. Nevertheless, even in these schemes, the silica Raman background remains a major drawback. In hollow-core fibres, light propagates in air and therefore interacts very little with the silica cladding. This results in a significant reduction of the generated silica background [16]. Recently, Yerolatsitis et al. showed that in a hollow-core negative-curvature fibre (NCF), the silica background Raman emission was at least 1000 times smaller than in a conventional solid fibre, while maintaining the same collection efficiency [17]. To realize a functional probe, the hollow core of the fibre needs to be end-capped to prevent any fluid from flowing inside the hollow core due to the capillary action effect. Previously, in [18], a fusion splicer was used to seal the end of a hollow-core PCF by arcing the end of the fibre. Although the capillary action problem was solved, this method leads to a poor collection efficiency. Alternatively, in [19], a silica bead was used to seal the hollow core. This only partially solves the problem, as it provides a seal for the hollow core but not the surrounding structure.

Here, we demonstrate the fabrication and characterization of a fibre probe comprising the hollow-core NCF presented in [17] and a spliced spherical micro-lensed fibre cap. The outer probe diameter was less than 250 µm. By splicing a micro-lensed fibre cap at the distal end of our fibre [17], we provided fibre closure, blocking the sample from entering the hollow parts of the fibre while improving the collection efficiency of the probe. Moreover, the combination of the NCF and micro-lens in one fibre probe provided a robust and miniaturized device ideal for in vivo Raman spectroscopy. The sensing capabilities of the micro-lensed fibre probe were demonstrated by measuring the Raman signal of different glucose concentrations in aqueous solutions. Glucose is a complex analyte that is clinically relevant but also difficult to measure [20]. 

## 2. Experimental

### 2.1. NCF and Probe Fabrication

**Figure 1:**
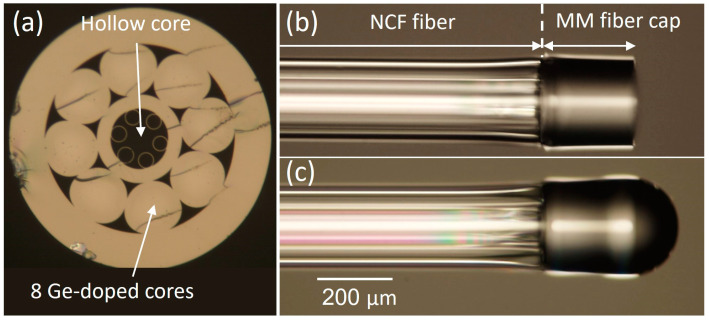
(**a**) The cross section of the NCF (the outer diameter of the fibre is 210 µm); (**b**) NCF probe with cleaved MM fibre; (**c**) NCF probe with spherical micro-lens.

The hollow-core NCF was fabricated using the stack-and-draw technique [16,17], and the cross section of the fibre is shown in Figure 1a. A more detailed description of the fibre design, fabrication, and characterization, including the transmission properties, can be found in [17]. The inner region consists of a single ring of six silica capillaries around a hollow core. The hollow core is designed to guide the 785 nm excitation light. In addition, surrounding the hollow-core, we introduced a ring of Ge-doped cores (peak NA = 0.3) designed to collect the Raman signal from a distal sample and therefore to increase the collection efficiency of the probe. 

The NCF probes were fabricated by splicing the NCF to a multimode (MM) fibre (FT200EMT, Thorlabs, Newton, NJ, USA) using a Fujikura FSM-100P fusion splicer. A robust splice without collapsing the hollow core of the NCF was ensured by reducing the splicing power, while the interface between the MM fibre and the NCF was shifted from the central axis of the splicer electrodes so that the NCF received less heat. Next, the MM fibre was cleaved to a 220 μm length (Figure 1b), creating a probe with a cleaved end-face. To create a lensed probe, the MM was cleaved to 340 μm, and the MM end was heated to its melting point while being rotated to minimize the influence of gravity using the Fibre Shaping mode in the Fujikura FSM-100P fusion splicer. This resulted in a spherically shaped end with a 240 μm diameter and low concentricity error (Figure 1c). The total lengths of the fabricated probes were 15 cm.

### 2.2. Experimental Setup

**Figure 2:**
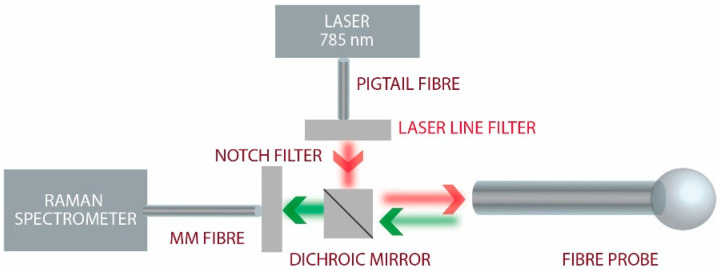
Experimental setup where light from a 785 nm laser is coupled to the NCF fibre probe using a dichroic mirror and collected Raman-shifted wavelengths are measured with a Raman spectrometer.

The experimental setup is presented in Figure 2. Light from a single-mode fibre pigtailed 785 nm diode laser (Thorlabs, Newton, NJ, USA) was coupled to the NCF via a dichroic mirror (LPD01-785RS-25, Semrock, West Henrietta, NY, USA). A bandpass filter (LL01-785-25, Semrock, West Henrietta, NY, USA) was used before coupling to the NCF, and a 791.6 long-pass filter (LP02-785RU-25, Semrock, West Henrietta, NY, USA) was introduced in the collection arm to filter out any residual pump. A multimode fibre with a graded-index core of 100 μm diameter (peak NA = 0.3) was used to collect the returned light from the entire proximal end-face of the NCF and deliver it to the fibre-coupled spectrometer (Avantes SensLine Hero, Apeldoorn, The Netherlands).

## 3. Results and Discussion

**Figure 3:**
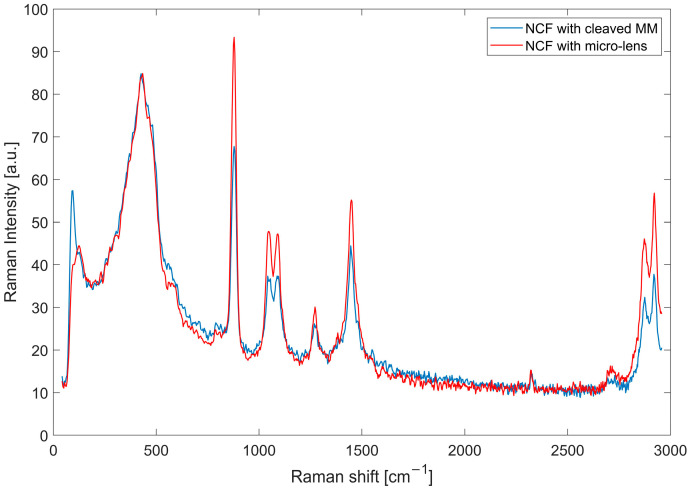
Collected Raman spectra of ethanol with the NCF probe with cleaved MM fibre and the NCF probe with the micro-lens. Note that the silica background around 500 cm^−1^ remains constant, whereas the ethanol peaks are enhanced by the micro-lens by a factor of 1.5.

The Raman scattering spectra of ethanol (Figure 3) were measured by immersing the distal ends of the probes in the sample. The spectra were taken with a 1 s integration time and five-times averaging.

**Figure 4:**
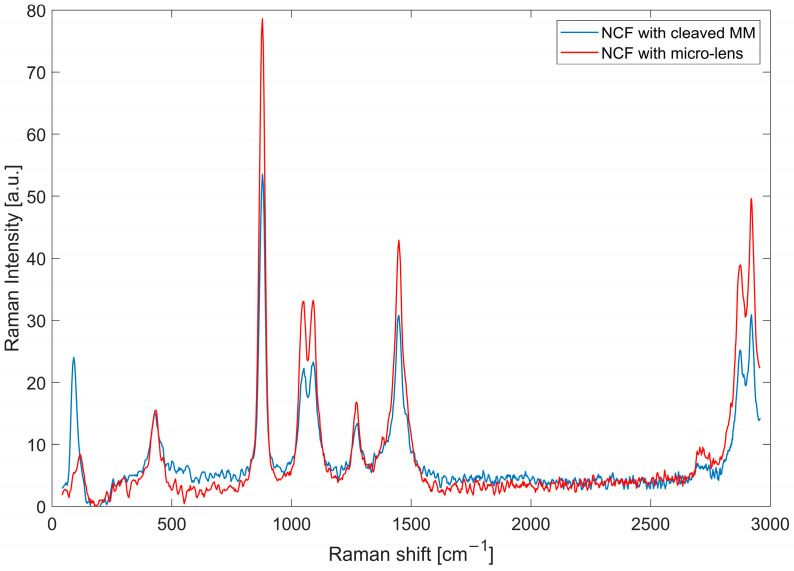
Ethanol spectrum after background removal and smoothing for a cleaved MM probe and a micro-lens probe.

The background spectrum measured in water was removed and a seventh-order Savitzky–Golay smoothing filter was applied (Figure 4). The result shows a significant Raman signal improvement when using the micro-lensed fibre. The 878 cm^−1^ peak has about 1.5-times higher intensity. Similar enhancements are seen for other lines. The higher Raman signal measured with the micro-lensed probe is related to two effects. The micro-lens focuses the excitation beam to a smaller volume, resulting in higher power density in the sample region within the volume in the collection fibre’s fields of view. 

Moreover, the micro-lens increases the numerical aperture (NA) of the probe. The micro-lens transforms the large angular distribution of the Raman scattering from the sample into a beam that falls within the NA of the collection cores. The NA of the micro-lensed probe was estimated to be about 0.7, compared to 0.22 for the MM fibre used for the cap.

**Figure 5:**
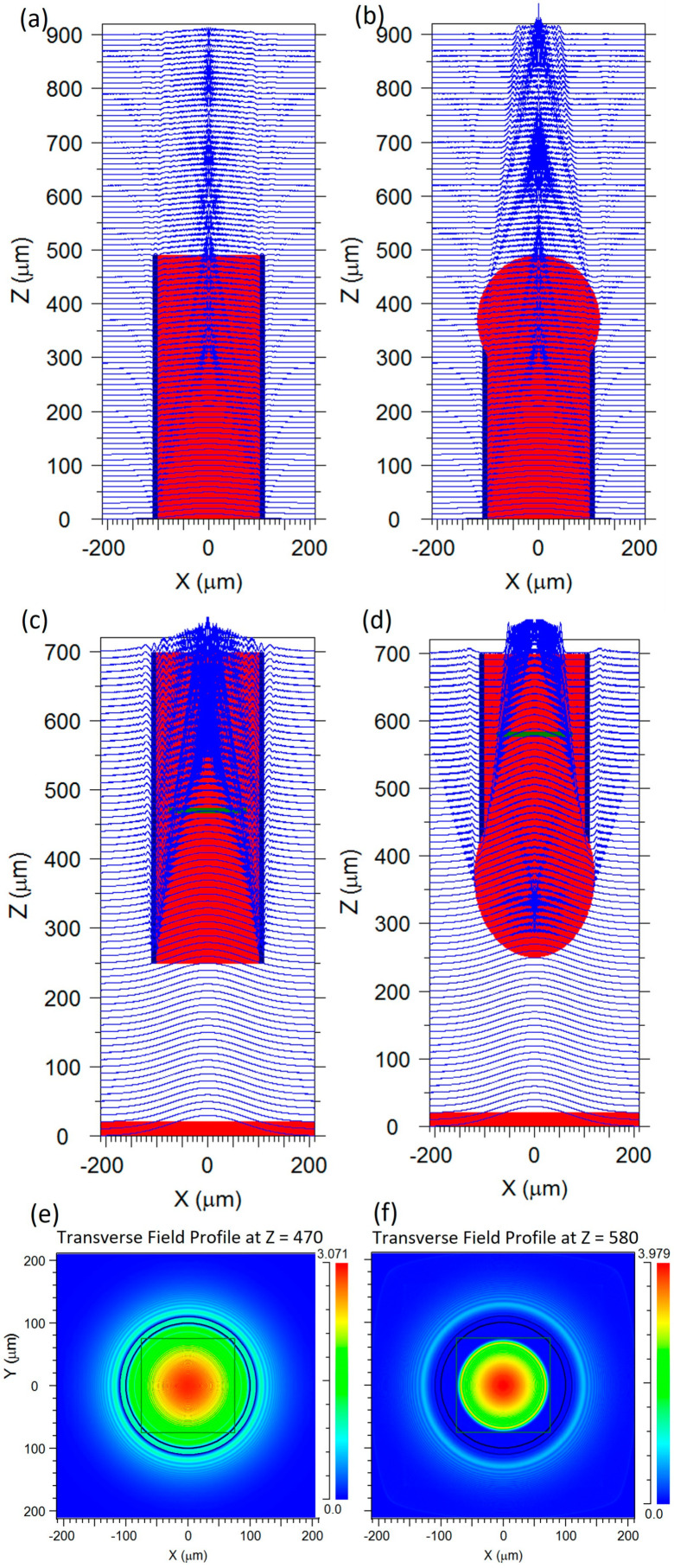
Simulated light propagation out of fibre probes, z-propagation direction, background refractive index 1.36: (**a**) cleaved MM fibre; (**b**) micro-lensed fibre. Simulated light coupling into: (**c**) MM fibre; (**d**) micro-lensed fibre. Transverse field profile at the splice position (green line in (**c**,**d**)) for the: (**e**) cleaved MM fibre; (**f**) micro-lensed fibre.

To illustrate the improved performance using a micro-lensed probe we used BeamPROP (RSoft, Synopsys, Mountain view, CA, USA) to simulate light propagation and coupling to a cleaved and a micro-lensed capped NCF. Figure 5a,b show the result using a 785 nm Gaussian beam excitation at z = 0. As expected, the micro-lens focuses the excitation beam into a smaller sample volume. The result is a larger number of Raman-shifted photons in the probe’s field of view. In Figure 5c,d the simulation has been reversed, using three Gaussian beams at z = 0, separated by 15 μm, to simulate the generated Raman signals. The light collected by the micro-lensed fibre was focused inside the fibre allowing for more light to be efficiently coupled back to the NCF and Ge-doped cores. 

A transverse field profile was also investigated at the splicing point with the NCF (green line in Figure 5c,d). The black square in Figure 5e,f represents the position of the outer cores of the NCF, verifying that the light collected and focused with the micro-lensed fibre matched the NCF cores, providing better light coupling. A rough estimate of the improvement in collection efficiency can be found by taking the ratio of the power within a disc with a radius equal to the outer radius of the eight Ge-doped cores. Unfortunately, the power monitor provided by the simulation software was limited to squares. However, since the observed power distribution for the cleaved fibre probe was almost uniform in the area between the square and the circle inscribed in the square, while the power in the corresponding area was zero for the micro-lensed fibre, we could estimate the improvement in collection efficiency by scaling the ratio of the power within the squares with a factor given by the ratio of the areas of the square and the circle. The power ratio was 1.24, while the scale factor was 1.27 (4/pi), resulting in an estimated improvement of 58%. This compares favourably with the observed 1.5-times increase in the collection efficiency of the collected Raman signal from our sample, as shown in Figure 4. 

**Figure 6:**
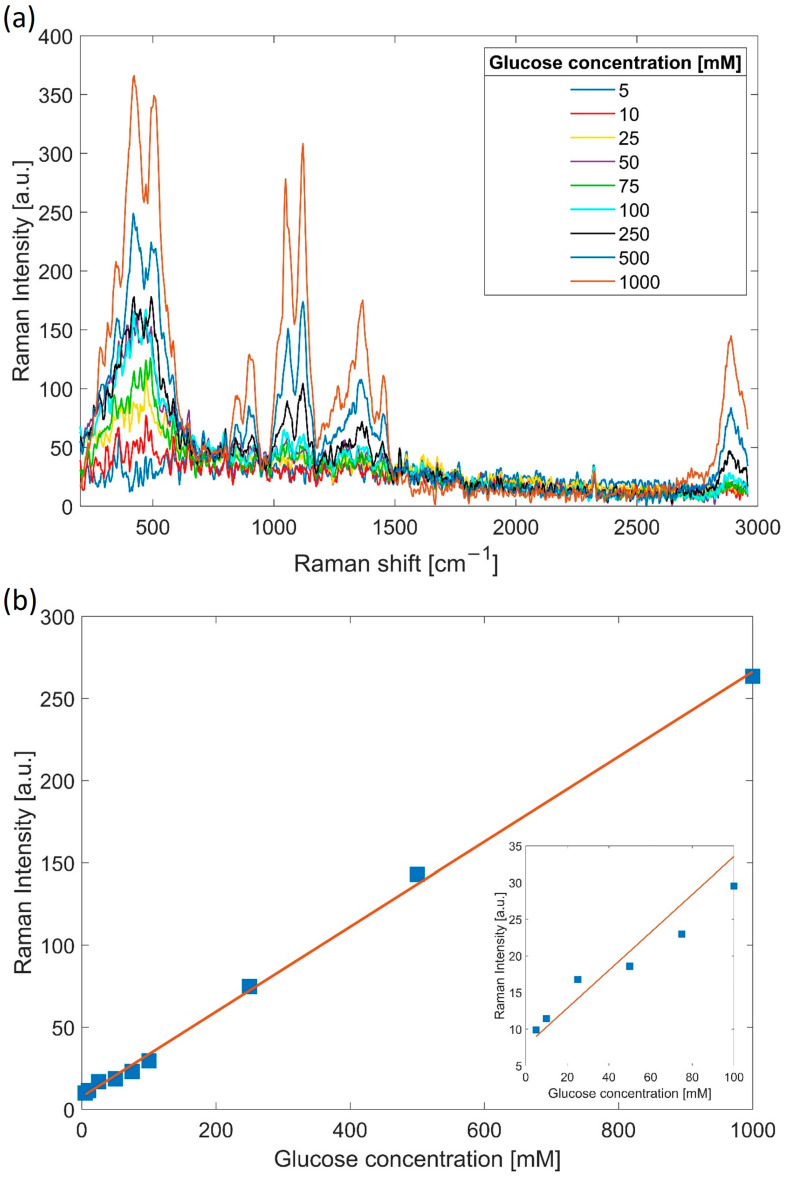
(**a**) Measured Raman spectra for different glucose concentrations; (**b**) 1120 cm^−1^ peak intensity as a function of the glucose concentration in mM (blue points) and linear fit (red). Insert: closeup of concentration range 5–100 mM.

For the glucose concentration measurements using the micro-lensed fibre probe, glucose powder (D-(+)-Glucose, Sigma-Aldrich, Burlington, MA, USA) was dissolved in ultrapure water (Millipore) and diluted to different concentrations in the range of 0 to 1 M. The NCF micro-lensed tip was immersed in the glucose solutions during the measurements and rinsed with water before each new concentration measurement to remove any residual glucose on the surface. The measurements were performed at room temperature, while the spectra were acquired with 60 s integration time. The Raman background signal measured with the pure water sample was subtracted from the spectra (Figure 6a). As before, the spectra were pre-treated with the Savitzy–Golay smoothing function (seventh order). The spectra clearly show increasing glucose-related Raman peaks with increasing glucose concentration. Although the use of the NCF fibre greatly reduced the silica background (by 1000×), it still contributed to the signal below 600 cm^−1^. Therefore, the 1120 cm^−1^ peak was selected for further analysis since it had the highest signal-to-noise ratio and it had been previously used by others to investigate glucose concentration [21,22]. For the analysis of the 1120 cm^−1^ peak versus glucose concentration, a simple baseline correction was applied by fitting a third-degree polynomial to the minima located between the Raman peaks in the 800–1500 cm^−1^ wavenumber region [15]. The intensity of the Raman signal for the 1120 cm^−1^ peak versus glucose concentration is presented in Figure 6b, showing a linear correlation in the measured concentration range. The measurements below 50 mM exhibit deviations from linearity related to a lower signal-to-noise ratio (Figure 6b insert). The limit of detection (LOD) was estimated at 50 mM, based on the 1120 cm^−1^ peak being 3 times higher than the noise level for this concentration. It is important to mention that glucose is difficult to measure with Raman scattering since the single Raman peaks are convoluted spectra of multiple vibrational modes [20]. 

## 4. Conclusions

We demonstrated an optical fibre probe for Raman spectroscopy based on a hollow-core negative-curvature fibre, incorporating an outer ring of eight large conventional multimode fibres, capped with a micro-lensed MM fibre. The hollow-core fibre was used to minimize the Raman background from the silica, while the micro-lensed cap blocked the sample from entering the hollow parts of the fibre and enhanced the collected Raman signal. The simple fabrication of the probe could lead to the realization of the practical use of hollow-core fibres for Raman in vivo sensing. The Raman signal was improved by 50% compared to that of a cleaved cap. Numerical simulation confirmed a good match of the light coupled back to the outer MM cores with the micro-lensed cap. The presented glucose concentration measurements show linear correlation between the Raman intensity and glucose concentrations, with the limit of detection being 50 mM. We believe that with further development of the system and the fibre, e.g., by reformatting the collection cores to a linear array of fibres to match the input slit of the spectrometer, we can increase the collection efficiency and therefore detect lower concentrations of complex analytes (e.g., glucose). In addition, even better performance and increased sensitivity is expected by combining the probe with surface-enhanced Raman scattering. This will also open up the prospect of using the probe in a range of applications such as environmental monitoring, food analysis, and virus detection and identification.

## Data Availability

Data underlying the results presented in the paper are available https://doi.org/10.15125/BATH-01100.
